# Disrupted functional network topology mediates the correlation between childhood trauma and aggression in youths with internet gaming disorder

**DOI:** 10.1093/braincomms/fcaf407

**Published:** 2025-10-16

**Authors:** Shijie Chen, Hongwei Wen, Yuejiao Zhang, Yuhong Zhou, Xuemei Gao

**Affiliations:** Faculty of Psychology, Southwest University, Chongqing 400715, China; Key Laboratory of Cognition and Personality, Ministry of Education, Southwest University, Chongqing 400715, China; School of Medical Imaging, North Sichuan Medical College, Nanchong, Sichuan 637000, China; Faculty of Psychology, Southwest University, Chongqing 400715, China; Key Laboratory of Cognition and Personality, Ministry of Education, Southwest University, Chongqing 400715, China; Psychological Research and Consultation Center, Southwest Jiaotong University, Chengdu 611756, Sichuan, China; Psychological Research and Consultation Center, Southwest Jiaotong University, Chengdu 611756, Sichuan, China

**Keywords:** internet gaming disorder, childhood trauma, aggression, functional connectivity network, graph theory

## Abstract

Aggression is a prevalent concern among adolescents with Internet Gaming Disorder (IGD), especially those with a history of childhood trauma. While IGD and childhood trauma are linked to aggression, the underlying neurobiological mechanisms remain poorly understood. This study aims to investigate how childhood trauma influences aggressive behaviour in adolescents with IGD through brain functional network alterations. A total of 108 adolescents with varying levels of IGD and childhood trauma were recruited and divided into IGD-with-trauma, IGD-without-trauma and healthy controls. Resting-state functional connectivity and graph theory analyses were used to investigate the global and nodal topological disruptions between groups. Then, correlation and mediation analyses were conducted to assess the relationship between functional network alterations, childhood trauma (Childhood Trauma Questionnaire–Short Form score) and aggression scores. Although all groups showed small-world topology in functional networks, compared to controls, both IGD groups exhibited significantly decreased normalized clustering coefficient (γ) and small-world index (σ). For regional topology, IGD with trauma group exhibited significantly reduced efficiency in bilateral superior parietal gyrus, left hippocampus, pallidum and thalamus compared with IGD without trauma group. Furthermore, γ, σ and nodal efficiency of left superior parietal gyrus not only showed significant correlations with Childhood Trauma Questionnaire–Short Form and Reactive Aggression scores, but also significantly mediated the correlation between Childhood Trauma Questionnaire and Reactive Aggression scores. These findings highlight both local brain dysfunctions and global topological disruptions contribute to aggressive behaviour, and provide valuable guidance for future intervention strategies that improving the integration and efficiency of brain functional networks may help reduce aggression in at-risk youths with IGD and trauma histories.

## Teaser

Chen *et al*. report that adolescents with Internet Gaming Disorder and childhood maltreatment show reduced brain network efficiency, particularly in the superior parietal region. These disruptions are associated with higher reactive aggression. The authors conclude that impaired attentional regulation may underlie aggressive behaviour in this population

## Graphical Abstract

**Graphical Abstract:**
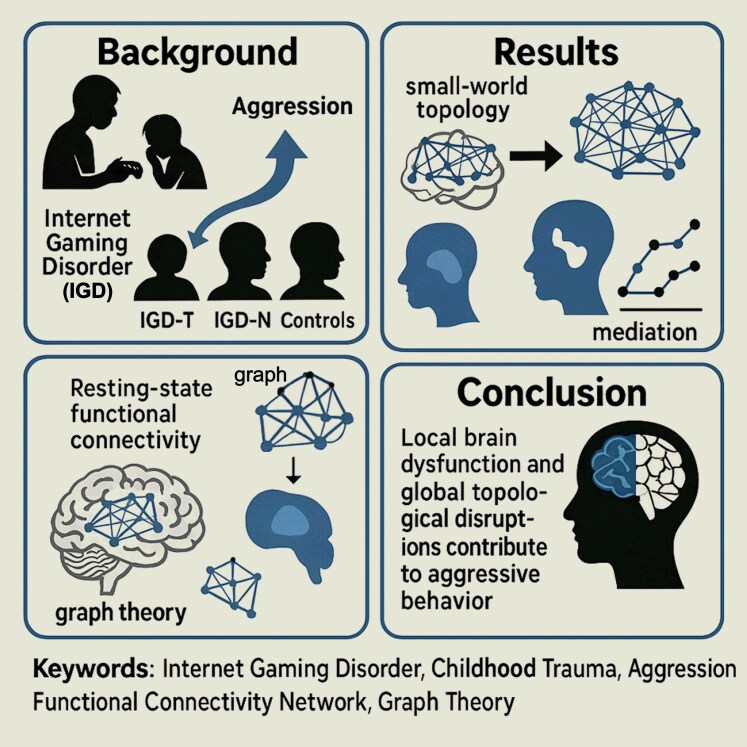

## Introduction

Internet Gaming Disorder (IGD) is a behavioural addiction characterized by persistent gaming despite adverse consequences. It is classified as a mental disorder in Section III of the Diagnostic and Statistical Manual of Mental Disorders, Fifth Edition^1^ and a behavioural addiction in the International Classification of Diseases (ICD-11, 2020). IGD significantly impacts various aspects of the lives of adolescents, including their social relationships, academic performance, and aggressive behaviours.^2-4^ Among its associated challenges, aggression is particularly concerning, especially in individuals with a history of childhood maltreatment. Such behaviour not only impairs emotional well-being^3^ but also poses significant societal challenges. Despite extensive research on IGD and aggression, the neurobiological mechanisms underlying this relationship, particularly when compounded by childhood trauma, remain poorly understood.

Emerging evidence shows that IGD and childhood trauma have independent effects on aggression but also that they may have a synergistic effect that further affects aggressive tendencies.^5,6^ Specifically, childhood maltreatment disrupts emotional regulation and impulse control,^7^ heightening susceptibility to stress-induced aggressive responses. It also promotes maladaptive coping strategies—such as gaming—to escape emotional distress, which can lead to IGD.^8^ Conversely, IGD itself reinforces aggressive tendencies developed in virtual contexts, often spilling over into real-world interactions.^4,9^ Adolescents with a history of childhood maltreatment are particularly vulnerable to developing IGD,^8^ creating a feedback loop that exacerbates aggressive behaviours. Consequently, childhood abuse not only has a direct effect on aggression but may also indirectly exacerbate it by accelerating IGD. Although these factors significantly influence aggressive behaviour, the extent to which the heightened aggression seen in youths with IGD can be linked to experiences of childhood abuse remains unclear. In addition, the neurobiological mechanisms by which childhood trauma affects aggression in youths with IGD—whether directly or indirectly— are still poorly understood, making it essential to explore these factors to better elucidate this complex relationship.

Previous studies utilizing functional magnetic resonance imaging (fMRI) technology have shown that both gaming addiction and childhood maltreatment correlate with functional alterations in brain regions responsible for emotional regulation, sensory processing, and cognitive control.^4,10^ For instance, some studies have found that individuals with gaming addiction exhibit significant decreases in spontaneous low-frequency fluctuations (ALFF) in regions such as the parietal lobe^11^ and thalamus,^12^ while individuals affected by childhood maltreatment also demonstrate similar functional alterations in these regions.^13^ Although these studies provide valuable insights into the functional changes in isolated regions, they lack a thorough exploration of interregional functional connectivity(FC), which is essential for understanding the broader neural networks involved. In fact, FC reflects the collaborative activity between different brain regions, offering a more comprehensive understanding of the neural mechanisms underlying emotional regulation and impulse control.^14^ Existing research suggests that gaming addiction and childhood trauma may lead to significant changes in FC. For example, individuals with gaming addiction often show reduced strength of connectivity between the prefrontal cortex and other emotion-regulation-related areas,^15,16^ and can even be further divided into two subgroups associated with differing patterns of brain FC and distinct clinical symptom profiles.^17^ Meanwhile, individuals with childhood trauma may exhibit unstable or heterogeneous functional connectivity patterns.^18^

Neurobiological evidence suggests that both IGD and childhood maltreatment disrupt critical brain regions involved in emotional regulation and sensory processing.^13,14,16,19^ Specifically, these disruptions affect areas associated with cognitive processing and memory functions,^16^ as well as those responsible for sensory integration and motor coordination.^13^ However, previous studies often focus on investigating FC changes between specific regions,^13,15,16,20^ neglecting the global network topological disruptions underlying behavioural dysregulation. To better capture these global disruptions, graph-theoretical analysis offers a valuable framework for examining brain networks.^21^ This approach explores topological properties such as small-world properties, network efficiency, and functional modularity, providing insights into how network topological alterations contribute to cognitive and emotional dysfunctions.^21,22^ Considering the combined effects of IGD and childhood maltreatment, a comprehensive analysis of brain network topology is essential. The interaction between these factors may disrupt global brain organization, intensifying aggressive behaviours and complicating emotional regulation. By applying graph-theoretical methods, we can uncover the underlying neurobiological mechanisms that drive these complex interactions, ultimately informing targeted interventions for affected individuals.

This study aimed to investigate the neurobiological basis of the impact of childhood maltreatment on aggression in adolescents with IGD from the perspective of functional network topology. Using resting-state fMRI and graph-theoretical methods, we sought to identify changes in the functional network topology of IGD adolescents (with or without childhood trauma) compared to healthy adolescents. We then aimed to correlate this altered network topology with levels of childhood trauma and aggression. Finally, mediation analysis was applied to clarify the neural basis through which childhood maltreatment affects aggression within the context of IGD. We hypothesized that (1) both IGD and childhood maltreatment increase the risk of aggressive behaviour by impacting cognitive function, emotional regulation, and reward regulation, resulting in disrupted functional network topology in the brain, and (2) that functional network topological biomarkers serve as potential mediating variables that amplify the impact of childhood trauma on aggressive behaviour in IGD.

## Materials and methods

### Ethics

The study was carried out following the Declaration of Helsinki. The Human Investigations Committee of Southwest University approved this research. All subjects were informed about the study and all provided informed consent.

### Participants

Participants were adolescents aged 12–18 years, comprising 30 who had IGD without trauma (IGD-N), 41 with IGD and trauma (IGD-T), and 37 healthy controls, recruited from the same school to ensure comparable educational backgrounds. IGD participants met DSM-5 criteria for IGD based on a structured clinical interview, while healthy controls were age-and gender-matched and scored below the IAT cutoff for addiction. Exclusion criteria included any history of neurological disorders, major psychiatric illness (e.g. mood disorders, schizophrenia), or current substance use. It is important to note that the participants were adolescents who had dropped out of school due to gaming addiction, as social and academic impairments are central to adolescent gaming addiction. We argue that this group accurately reflects the population of adolescents with IGD, and their inclusion improves the study’s representativeness.

### Study measures

To enhance response accuracy and reduce bias, trained research staff read each item aloud, clarified any questions, and emphasized that there were no ‘right’ or ‘wrong’ answers. Questionnaires were administered in a quiet, private setting, and participants were assured of the anonymity of their responses to minimize social desirability effects.

*Internet Addiction Test and DSM-5 Criteria for IGD:* The Internet Addiction Test (IAT) includes 20 items, each scored on a 5-point Likert scale (1 = rarely, 5 = always). A score over 50 indicates addiction. The IAT has been widely validated and used in prior IGD studies.^23,24^ Additionally, a structured clinical interview was conducted to assess whether participants met the DSM-5 criteria for IGD, with addiction being defined as meeting at least five of the nine criteria.^1^ Participants in this study were screened using the following criteria: (1) an IAT score above 50 and (2) meeting at least five DSM-5 criteria. Healthy controls were defined by (1) an IAT score below 50 and (2) meeting fewer than five DSM-5 criteria.

*Childhood Trauma Questionnaire–Short Form (CTQ–SF):* the CTQ*–*SF contains 28 items, divided into five subscales: emotional abuse, physical abuse, sexual abuse, emotional neglect, and physical neglect. Each item is rated on a 5-point scale (1 = never true, 5 = very often true), with subscale scores ranging from 5 to 25. Higher scores indicate more severe maltreatment. The CTQ*–*SF is a widely used, reliable measure for assessing childhood abuse and neglect.^25^ For defining moderate to severe childhood trauma, we used the following criteria based on subscale scores: (1) emotional abuse ≥ 13, (2) physical abuse ≥ 10, (3) sexual abuse ≥ 8, (4) emotional neglect ≥ 15, or (5) physical neglect ≥ 10. Participants not meeting any of these thresholds were considered trauma-free. Due to sample size limitations, we did not further subdivide the IGD-T group by specific trauma subtype.

*Reactive-Proactive Aggression Questionnaire (RPQ):* the RPQ consists of 20 items rated on a 3-point scale (0 = never, 1 = sometimes, 2 = often), with 10 items measuring reactive aggression (RA) and 10 items measuring proactive aggression (PA). The total score is the sum of responses to both sets of items. The RPQ has been widely used and validated for assessing aggression in children and adolescents^26^

### Image acquisition

MRI data were acquired on a Siemens Prisma 3.0T scanner (Siemens, Erlangen, Germany) using a 64-channel head/neck coil. Prior to scanning, participants were instructed to lie supine. They wore noise-cancelling headphones and were reminded to keep their head fixed, eyes closed, and to remain physically and mentally relaxed during data acquisition. Resting-state blood oxygen level-dependent (BOLD) fMRI data were acquired using a simultaneous multi-slice echo-planar imaging sequence with the following parameters: repetition time (TR) = 2000ms, echo time (TE) = 30 ms, field of view (FOV) = 224 × 224 mm^2^, matrix size = 112 × 112, slice thickness = 2 mm, slice gap = 2.3 mm, 62 axial slices, and 240 time points. High-resolution brain structural images were acquired via a T1-weighted 3-dimensional magnetization prepared rapid gradient echo (MPRAGE) sequence with the following parameters: TR/TE = 2530/2.98 ms, inversion time = 1100 ms, flip angle = 7, slice thickness = 1 mm, FOV = 256 × 224 mm^2^, and matrix size = 512 × 448.

### Image preprocessing

The acquired resting-state fMRI and T1-weighted images were preprocessed using the Data Processing & Analysis for Brain Imaging Toolbox (DPABI v 6.0, http://rfmri.org/dpabi) as follows: 1) removing the first 10 volumes of each functional scan; 2) slice timing and head motion correction, with participants being excluded if their maximum rotation was more than 3.0°, maximum translational displacement was more than 3.0 mm, or mean frame-wise displacement (FD) was more than 0.2 mm^27^; 3) co-registering the T1w image to the mean functional image; 4) segmenting the T1w images into grey matter (GM), white matter (WM), and cerebrospinal fluid (CSF) using the New Segment tool; 4) normalizing the segmented GM images to MNI standard space to obtain deformation information using the DARTEL algorithm^28^; 5) nuisance signals, including CSF, WM, and Friston-24 head motion parameters^29^, were regressed out for functional images; 6) the deformation fields derived from corresponding GM images were used to normalize the functional images to MNI space; 7) normalized images were resampled to 3-mm cubic voxels; 8) spatially smoothed with a Gaussian kernel (full width at half maximum = 4 mm); 9) bandpass filtering (0.01–0.1 Hz).

### Constructing functional connectivity networks and graph theory analysis

After image preprocessing, a total of 90 regions of interest (ROIs) from the automated anatomical labelling (AAL) atlas were used as nodes (shown in Supplementary Table 1) and classified into five functional modules.^30^ Then, the BOLD signals for each node were averaged, and Pearson correlation coefficients were calculated to determine the correlation between BOLD signals across nodes, resulting in a 90 × 90 symmetric functional connectivity network.

Using the graph theoretical network analysis toolbox GRETNA (GRETNA, http://www.nitrc.org/projects/gretna/),^31^ we calculated network topological measures across sparsity levels ranging from 8% to 60% at 2% intervals, consistent with our previous studies.^32,33^ This range ensured the network remained fully connected at the lower threshold while preventing excessive randomization and loss of biological plausibility beyond the upper threshold.^34^ Seven global topological metrics were calculated: global efficiency (E_glob_), local efficiency (E_loc_), shortest path length (L_p_), clustering coefficient (C_p_), normalized L_p_ (λ), normalized C_p_ (γ), and small-worldness (σ). For regional topological metrics, we considered nodal efficiency (E_nodal_). Supplementary Table 2 shows the general introductions for the network metrics. As the network metrics were calculated for each sparsity threshold, we also calculated the area under the curve (AUC) for each network metric across sparsity levels to provide a summarized scalar independent of single threshold selection.

### Statistical analysis

For demographic and behavioural parameters, one-way analysis of variance (ANOVA) and least significant difference (LSD) post-hoc tests were used to assess differences in age, head motion, addiction, aggression, and trauma scores between groups, with the chi-square test used to analyse the sex ratio. For network topological metrics, one-way analysis of covariance (ANCOVA) and LSD post-hoc tests were used to identify differences between groups after controlling age, gender, and head motion (mean FD) as covariates, using SPSS software (v23.0, SPSS Inc., Chicago, IL).

Furthermore, once significant differences in any network metrics were identified among the three groups, the pairwise partial correlations between significant altered network metrics, aggression, and CTQ-SF scores were calculated for all IGD participants using age, gender, head motion, and IAT score as covariates. The significance threshold for correlation analysis was set to *P* < 0.05, with the false discovery rate (FDR;^35^ corrected for multiple comparisons.

Finally, mediation analysis was conducted to examine whether brain functional network biomarkers could modulate the relationship between childhood trauma and aggression in IGD participants. Briefly, we treated CTQ–SF score as an independent variable, PA or RA score as the dependent variable, significant altered network metrics as the mediating variable, and age, gender, head motion, and IAT score as covariates. We employed the simple mediation model (model 4) in Hayes’s Process macro^36^ for SPSS v. 21.0 software (SPSS Inc., Chicago, IL), incorporating a bootstrapping procedure with 5000 resamples to generate estimates and confidence intervals (CIs). Bootstrapping is a nonparametric resampling strategy^37^ that aids in estimating and testing indirect effects against zero by creating CIs. An indirect effect was deemed significant if the bootstrapped 95% CIs did not encompass zero.^37,38^ A classical effect size measure (effect proportion;^39^ for the mediation model was then calculated.

## Results

### Demographic and behavioural characteristics

There were no significant differences in age among the three groups, and although the sex distribution was imbalanced (more males in IGD groups), this reflects the known male predominance in IGD (8.5% versus 3.5%, 2.4-fold difference). The IGD-T group had significantly higher IAT, RA, PA, and CTQ-SF scores than both IGD-N and control groups, and the IGD-N group had higher IAT and RA scores than controls. Head motion (FD_Jenkinson) differed only between the IGD-N and IGD-T groups (Table 1). Importantly, after including age and gender as covariates, all primary group differences in network metrics and their associations with aggression remained significant (*P* < 0.05).

**Table 1:** Demographic and behavioural characteristics for each group

Characteristic	IGD-N	IGD-T	HC	*P* value (ANOVA/χ^2^)	*P* value (LSD post-hoc)		
					IGD-N versus HC	IGD-N versus IGD-T	IGD-T versus HC
Gender	23M/7 F	21M/20 F	26M/11 F	0.059^χ2^	-	-	-
Age (y)	13.93 ± 1.23	14.41 ± 1.28	14.43 ± 1.76	0.295^a^	-	-	-
IAT	63.67 ± 10.06	69.39 ± 11.68	36.00 ± 7.76	**<0**.**001**^a^	**<0**.**001**	**0**.**019**	**<0**.**001**
CTQ-SF	35.67 ± 5.70	62.46 ± 16.71	34.32 ± 7.76	**<0**.**001**^a^	0.640	**<0**.**001**	**<0**.**001**
PA	2.00 ± 1.84	4.51 ± 3.68	0.84 ± 1.12	**<0**.**001**^a^	0.066	**<0**.**001**	**<0**.**001**
RA	10.17 ± 3.47	13.54 ± 3.18	6.65 ± 3.47	**<0**.**001**^a^	**<0**.**001**	**<0**.**001**	**<0**.**001**
FD_Jenkinson	0.07 ± 0.03	0.05 ± 0.02	0.06 ± 0.0.03	**0**.**008**^a^	0.053	**0**.**002**	0.219

Notes: ^χ2^: chi-square test.

^a^: ANOVA test. FD_Jenkinson: framewise displacement^27^; IGD-N: internet gaming disorder without trauma; IGD-T: internet gaming disorder with trauma; HC: Healthy control group; IAT: Total addiction score; CTQ-SF: Childhood trauma questionnaire score; PA: Proactive aggression score; RA: Reactive aggression score. The bolding is used to highlight statistical significance.

### Aberrant global topology of functional networks between groups

**Figure 1:**
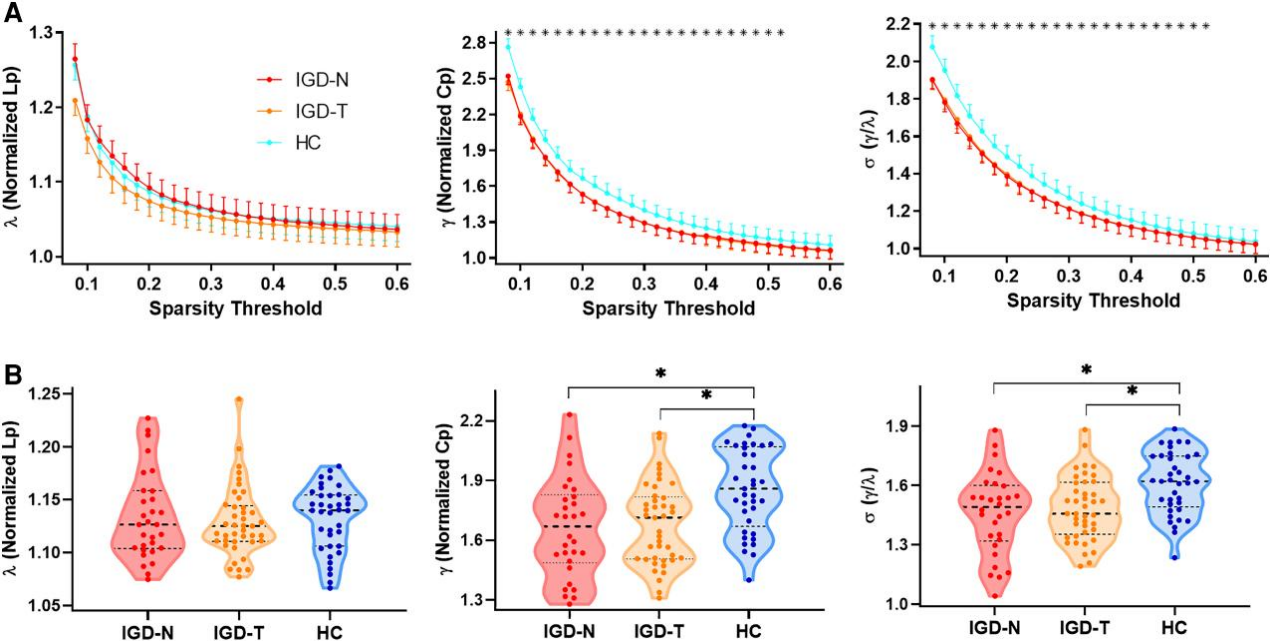
**Group comparisons of global topological properties.** (**A**)For each network metric, values across a range of thresholds are plotted for each group. Each data point represents the metric value for one participant at the corresponding threshold, and each asterisk represents a significant difference (*P* < 0.05, ANCOVA with post-hoc tests) in IGD-N (n = 30) and IGD-T (n = 41) groups compared with the control group (n = 37) for this network metric. (**B**) The violin plots represent the distribution of network metric in each group. Each data point represents one participant’s AUC value of network metrics, and the asterisk represents a significant difference (*P* < 0.05, ANCOVA with post-hoc tests) between two groups. Abbreviations: Lp: characteristic path length; Cp: clustering coefficient; HC: healthy controls; IGD-N: Internet Gaming Disorder without childhood trauma; IGD-T: Internet Gaming Disorder with childhood trauma.

Over the entire sparsity range, all groups exhibited the small-world topology characterized by γ>1, λ≈1 and σ=γ/λ>1 (Fig. 1A). Furthermore, we found that the IGD-N and IGD-T groups showed significantly decreased λ and σ compared with control group within a wide range of sparsity thresholds (Fig. 1A) and integrated AUC values (Fig. 1B and Table 2). No significant difference was observed in any network metric between IGD-N and IGD-T groups.

**Table 2:** Group comparisons of global topological properties of functional networks

Metrics (AUC value)	IGD-N	IGD-T	HC	*P* value (ANCOVA)	*P* value (post hoc)		
					IGD-N versus HC	IGD-N versus IGD-T	IGD-T versus HC
E_glob_	0.201 ± 0.005	0.193 ± 0.005	0.195 ± 0.007	0.423	-	-	-
E_loc_	0.270 ± 0.007	0.258 ± 0.007	0.261 ± 0.009	0.390	-	-	-
L_p_	1.396 ± 0.036	1.454 ± 0.037	1.438 ± 0.047	0.415	-	-	-
C_p_	0.223 ± 0.007	0.212 ± 0.007	0.216 ± 0.009	0.388	-	-	-
λ	1.132 ± 0.007	1.128 ± 0.007	1.135 ± 0.009	0.852	-	-	-
γ	1.640 ± 0.043	1.658 ± 0.044	1.859 ± 0.056	**0**.**024**	**0**.**007**	0.731	**0**.**022**
σ	1.436 ± 0.034	1.434 ± 0.035	1.617 ± 0.044	**0**.**015**	**0.005**	0.957	**0.008**

Notes: IGD-N: internet gaming disorder without trauma; IGD-T: internet gaming disorder with trauma; HC: Healthy controls. The bolding is used to highlight statistical significance.

### Aberrant nodal efficiency of functional networks between groups

Five brain regions exhibiting significantly (ANCOVA tests, *P* < 0.05) altered nodal efficiency were identified among three groups (Table 3). Furthermore, LSD post-hoc tests indicated the IGD-T group showed significantly decreased nodal efficiency in the bilateral superior parietal gyrus (SPG), left hippocampus, pallidum and thalamus compared with IGD-N group (Table 3). In addition, compared with controls, the IGD-T group showed significantly decreased nodal efficiency in the left hippocampus.

**Table 3:** Brain regions showing significantly altered nodal efficiency in functional networks between groups

		E_nodal_			*P* value (ANCOVA)	*P* value (post hoc)		
Module	Region	IGD-N	IGD-T	HC		IGD-N versus HC	IGD-N versus IGD-T	IGD-T versus HC
Subcortical	HIP.L	0.160 ± 0.026	0.146 ± 0.035	0.163 ± 0.030	**0**.**041**	N.S.	**0**.**047**	**0**.**022**
Subcortical	PAL.L	0.185 ± 0.040	0.164 ± 0.038	0.175 ± 0.037	**0**.**032**	N.S.	**0**.**011**	N.S.
Subcortical	THA.L	0.194 ± 0.048	0.157 ± 0.051	0.176 ± 0.041	**0**.**016**	N.S.	**0**.**006**	N.S
Sensorimotor	SPG.L	0.201 ± 0.042	0.173 ± 0.041	0.185 ± 0.040	**0**.**009**	N.S.	**0**.**006**	N.S.
Sensorimotor	SPG.R	0.195 ± 0.038	0.173 ± 0.042	0.182 ± 0.038	**0**.**045**	N.S.	**0**.**019**	N.S.

Abbreviations: E_nodal_ represents the AUC values (mean ± SD) of the nodal efficiency for each group; IGD-N: internet gaming disorder without trauma; IGD-T: internet gaming disorder with trauma; HC: Healthy controls, N.S., not significant. The modular division of brain regions was based on a previous study.^30^ For abbreviations of region, see supplementary Table 1. The bolding is used to highlight statistical significance.

### Correlation analysis results

**Figure 2:**
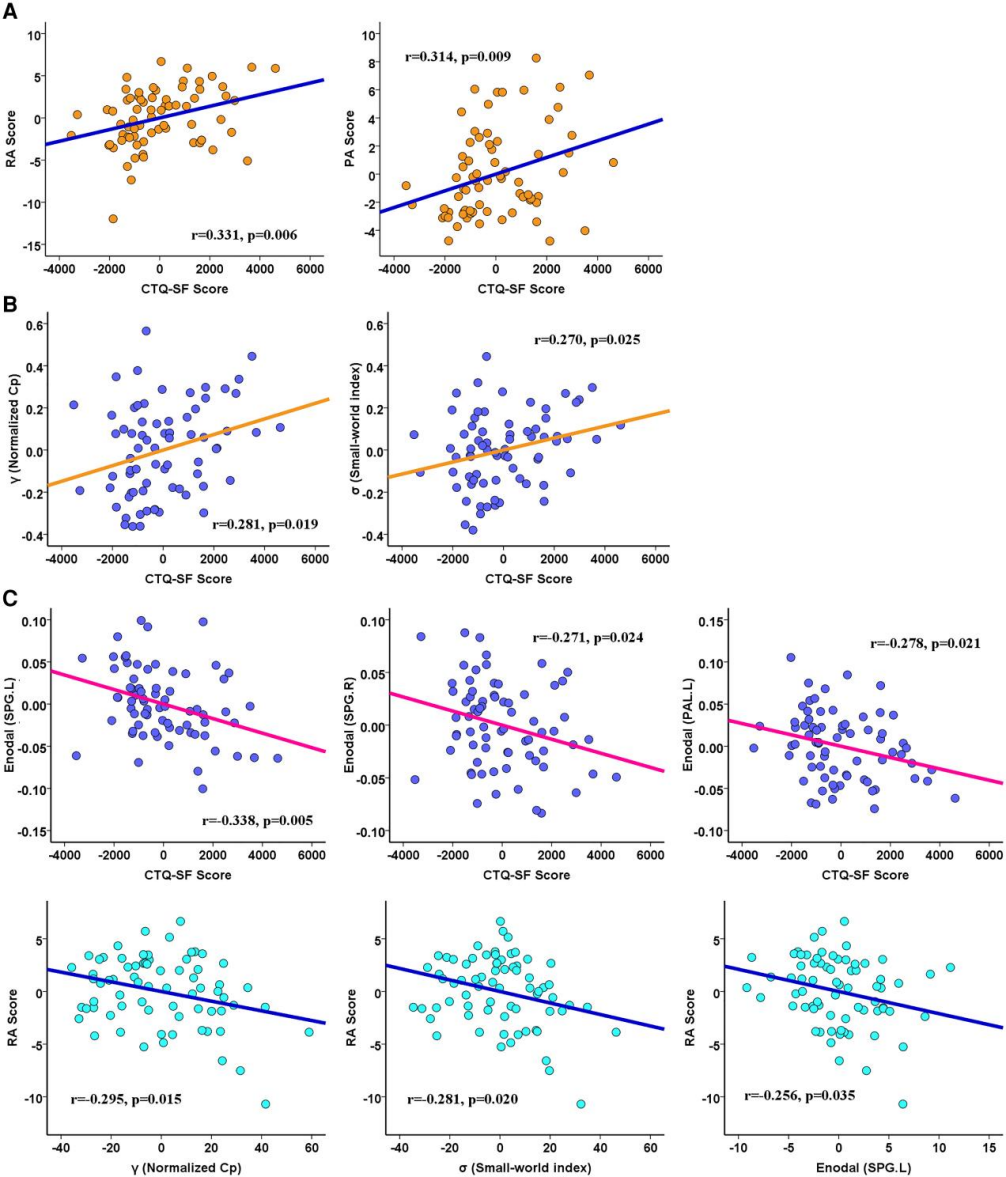
**Partial correlations among trauma, aggression, and network metrics.** Pairwise partial correlations among childhood trauma, aggression, and network metrics in all IGD subjects (including IGD-N subjects, n = 30; and IGD-T subjects, n = 41). The scatter plots show partial correlations between (**A**) CTQ-SF score and RA score, (**B**) network metric (mediator M) and CTQ-SF score, and (**C**) network metric and RA score. Each dot represents one IGD subject's residual score. Notably, the values of individual data points displayed on the X-axis and Y-axis reflect the residual values of these variables rather than their initial values, while considering age, gender, IAT score and head motion as covariates in partial correlations. Abbreviations: CTQ-SF: Childhood Trauma Questionnaire–Short Form; RA: Reactive Aggression; PA: Proactive Aggression; SPG.L: left superior parietal gyrus; SPG.R: right superior parietal gyrus; PAL.L: left lenticular nucleus, pallidum; Cp: clustering coefficient.

By calculating pairwise partial correlations between altered network metrics, aggression and childhood trauma scores in IGD subjects, after FDR corrected for multiple comparison, we found the RA (r = 0.331, *P* = 0.006) and PA (r = 0.314, *P* = 0.009) scores showed significant positive correlations with CTQ-SF score. In addition, the γ (r = 0.281, *P* = 0.019), σ (r = 0.270, *P* = 0.025) and nodal efficiency of bilateral SPG (r = −0.338, *P* = 0.005 and r = −0.271, *P* = 0.024) and left pallidum (r = −0.278, *P* = 0.021) showed significant correlations with CTQ-SF score (shown in Fig. 2). Meanwhile, RA score was significantly negatively correlated with γ (r = −0.294, *P* = 0.015), σ (r = −0.281, *P* = 0.020) and nodal efficiency of left SPG (r = −0.256, *P* = 0.035).

### Mediation analysis results

**Figure 3:**
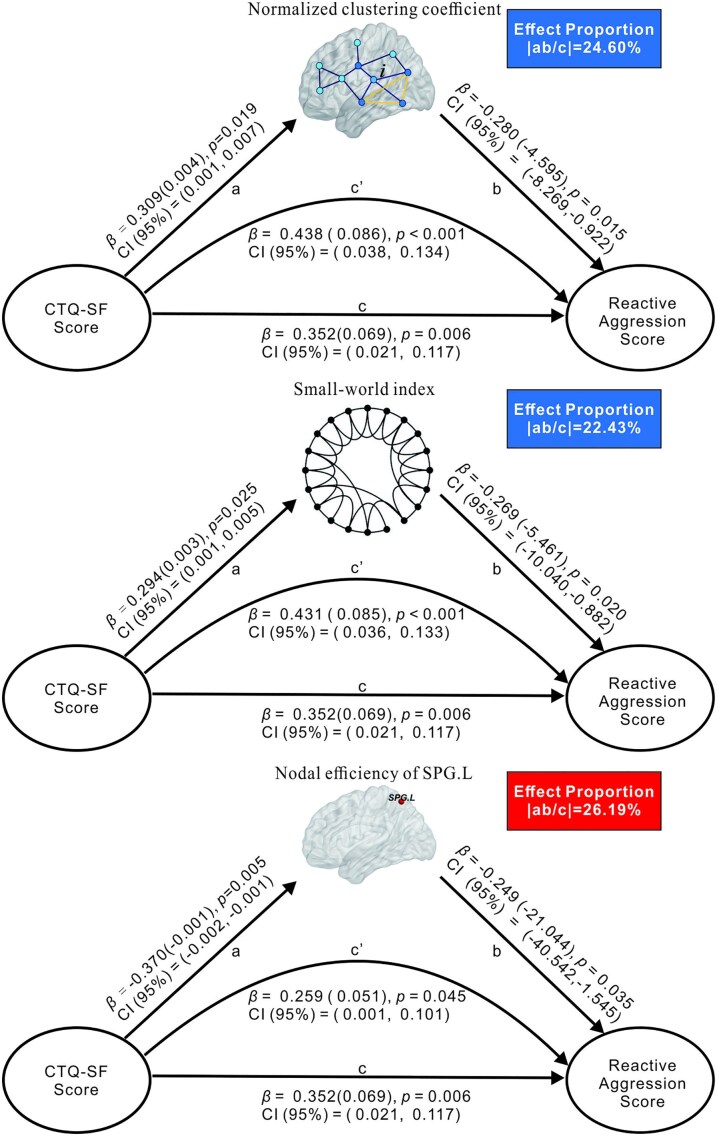
**Mediation model linking childhood trauma to reactive aggression.** Simple mediation analysis results for all IGD subjects (including IGD-N subjects, n = 30; and IGD-T subjects, n = 41). This model tests whether a network metric (mediator M) explains the relationship between CTQ-SF score and reactive aggression score. Note, ab: indirect effect; c’: direct effect; c: total effect; β(): β represents standardized regression coefficients, with unstandardized coefficients in bracket; CI(95%): 95% confidence intervals from 5000 bootstrapping iterations. Abbreviations: CTQ-SF = Childhood Trauma Questionnaire–Short Form; SPG.L: left superior parietal gyrus.

The simple mediation model showed significant mediation by the presence of brain functional network topology for childhood trauma in relation to reactive aggression for IGD subjects, after controlling age, gender, head motion and IAT score as covariates. In detailed (Fig. 3), we found that the nodal efficiency of left SPG partially mediates (effect proportion of 26.19%) the correlation between CTQ-SF and RA scores, where effect proportion equals as the absolute value of ratio of indirect effect to total effect. In addition, the correlation between CTQ-SF and RA scores was significantly mediated by γ (effect proportion of 24.60%) and σ (effect proportion of 22.43%), and due to the indirect and direct effects showing opposite signs (detailed shown in Table 4), we concluded them as masking effects.^40^

**Table 4:** Mediating effects of brain neural activity on relations of childhood trauma (CTQ-SF) with reactive aggression

Mediator	Effect Type	Dependent variable	Effects	Effect Value	Boot SE	Boot LLCI	Boot ULCI	Effect Ratio
γ (normalized clustering coefficient)	Masking effect	RA score	Indirect effects	−0.017	0.010	−0.038	−0.001	24.60%
			Direct effect	0.086	0.024	0.038	0.134	–
			Total effect	0.069	0.024	0.021	0.117	–
σ (small-world index)	Masking effect	RA score	Indirect effects	−0.016	0.008	−0.032	−0.001	22.43%
			Direct effect	0.085	0.024	0.036	0.133	–
			Total effect	0.069	0.024	0.021	0.117	–
Nodal efficiency of SPG.L	Partial mediation	RA score	Indirect effects	0.018	0.013	0.001	0.051	26.19%
			Direct effect	0.051	0.025	0.001	0.101	–
			Total effect	0.069	0.024	0.021	0.117	–

Notes: SPG.L: left superior parietal gyrus; CTQ-SF: Childhood trauma questionnaire score; RA: reactive aggression. Boot SE, bootstrap standard error; Boot LLCI, bootstrap lower limit 95% confidence interval; Boot ULCI, bootstrap upper limit 95% confidence interval. Effect ratio: the absolute value of the ratio of the indirect effect to the total effect.

## Discussion

To the best of our knowledge, this study is the first to investigate—from the perspective of brain functional network topology—the neural mechanisms by which childhood abuse influences aggression in adolescents with IGD. Our findings include: (1) significantly lower small-world index and normalized C_p_ were found in both IGD groups compared to the healthy controls, indicating impaired integration and efficiency in brain functional networks; (2) childhood abuse led to significantly reduced node efficiency of the bilateral superior parietal gyrus (SPG), left hippocampus, pallidum, and thalamus; (3) although childhood abuse significantly contributes to both PA and RA, altered functional network topological properties were only significantly correlated with RA; and (4) two small-world parameters and nodal efficiency of the left SPG mediated the relationship between childhood abuse and aggression in IGD adolescents.

### Global topological alterations in functional networks between groups

Our study identified that the global topological changes in brain functional networks caused by IGD are mainly characterized by two small-world parameters. We found that both IGD groups showed significantly decreased γ and σ compared to healthy controls, indicating a less efficient global brain network organization. Specifically, while γ reflects the density of local connections relative to a random network, indicating higher local clustering and processing efficiency, σ combines both local and global efficiency, highlighting the effective information flow between modules that enhances overall functional integration. Therefore, the decreased γ and σ in IGD groups suggest both local disorganization and impaired global communication within the brain network. These findings are consistent with previous research showing that IGD is associated with disrupted global network integration and modularity,^21,22,41^ reflecting impaired network efficiency that is commonly observed in individuals with behavioural addictions.^42,43^

However, we did not observe significant differences between the IGD-N and IGD-T groups in these global topological properties, which suggests that childhood abuse does not significantly exacerbate the global network alterations seen in IGD. This finding is in contrast to some studies that suggest childhood maltreatment can disrupt global brain network organization, leading to further inefficiencies in brain connectivity.^44,45^ Our results imply that the effects of childhood maltreatment might be localized and region-specific rather than producing additional global network disruptions. This pattern could reflect a different mechanism for how trauma influences brain function, which may not directly impact global organization but instead alters specific regions critical for emotional regulation and impulse control.

### Group differences in nodal efficiency of the functional network

For regional topology, our findings revealed significantly decreased nodal efficiency in the bilateral SPG in the IGD-T group than in the IGD-N group. The SPG plays a crucial role in sensory processing, spatial awareness, motor coordination, and cognitive control, all of which are critical for integrating sensory information and coordinating actions.^46^

Classical neurobiological models posit that aggression primarily involves a prefrontal-limbic circuit mechanism, wherein the dorsolateral and orbitofrontal cortices exert top-down inhibitory control over limbic structures (e.g. amygdala) to regulate impulsive responses. The bilateral superior parietal gyrus (SPG)—a core node of the dorsal attention network—contributes to this process not only through visuospatial attention and sensorimotor integration but also via attentional resource allocation. When SPG nodal efficiency is reduced, it impairs both the filtering of emotionally salient cues and the integration of contextual-sensorimotor signals. This dual deficit weakens prefrontal modulation of limbic reactivity, ultimately leading to failed cognitive control over impulsive behaviours, particularly difficulties in suppressing reactive aggression. This framework is empirically supported by findings that parietal dysfunction in aggression-prone individuals consistently correlates with attentional control deficits^47^and emotion dysregulation.^48^

In addition, we also found that the childhood abuse factor significantly reduced the nodal efficiency of the left hippocampus, pallidum, and thalamus. These regions are vital for memory processing, sensory integration, and emotional regulation.^21,41,49^ The hippocampus is particularly involved in the regulation of emotional memories, while the pallidum and thalamus play essential roles in integrating sensory information and controlling motor functions. Childhood trauma has been shown to impair hippocampal function and disrupt communication between it and other areas.^50^ Our findings suggest that childhood abuse exacerbates dysfunction in these regions, leading to further difficulties in emotional regulation and associated with higher levels of reactive aggression among IGD adolescents. Notably, the dysfunction observed in this study was associated with childhood abuse rather than IGD itself.

### Correlation analysis: brain network parameters and aggression

In the current study, we found that altered topological properties in the functional network were significantly correlated with RA but not with PA. This suggests that network changes in the brain, particularly in regions involved in sensory processing and emotional regulation (e.g. the SPG), may specifically influence RA, which is closely linked to emotional dysregulation and impulsivity. In RA, individuals often react impulsively to perceived threats or frustrations, which may involve heightened sensitivity in regions responsible for processing emotional stimuli.^51^ In contrast, PA is more goal-directed and planned and thus likely involves neural mechanisms related to executive control.^51^ These regions were less impacted in our study, indicating that childhood trauma may primarily disrupt emotional regulation and impulse control, influencing RA but not PA.

Intriguingly, the nodal efficiency of the left pallidum is significantly correlated with the CTQ–SF score. Despite not emerging as a significant mediator in the subsequent mediation analysis, its strong association with childhood trauma suggests that this region plays a critical role in the relationship between early-life adversity and aggression. The left pallidum is involved in reward processing and emotional regulation,^52^ both of which are critical for the modulation of aggression. Future research could explore how dysfunction in the left pallidum contributes to the emotional dysregulation seen in trauma-exposed individuals with IGD and whether interventions targeting this region might help mitigate emotional and aggression-related behaviours in these individuals.

### Mediation analysis: unpacking the mechanisms of aggression

The mediation analysis revealed compelling evidence for the mediating role of brain network topological properties in the relationship between childhood trauma and RA. Specifically, the analysis revealed that γ, σ, and the nodal efficiency of the left SPG significantly mediated this relationship. This finding suggests that alterations in these brain network features, particularly in regions involved in cognitive control and emotional regulation, are associated with the heightened aggression observed in adolescents with both IGD and childhood maltreatment.

Importantly, the efficiency of the left SPG acted as a partial mediator, indicating that childhood abuse may be linked to increased aggression via its effects on brain network organization. The SPG is a key region involved in spatial reasoning, working memory, and sensory integration, which are all cognitive functions that are often impaired in both IGD and trauma.^5,10,19,23^ The partial mediation effect of the left SPG suggests that deficits in these cognitive functions contribute to difficulties in emotional regulation and impulse control,^4,5,7,10^ which, in turn, lead to increased aggression. This process highlights the importance of cognitive and sensory processing networks in modulating aggressive behaviours in IGD individuals with childhood maltreatment histories, where dysfunction in these areas can result in heightened emotional reactivity and poor behavioural control.^5,7,22,53^

In contrast to the left SPG, γ and σ exhibited masking effects in the mediation analysis. This interesting finding suggests that the disruptions of small-world properties in functional networks may obscure the direct relationship between childhood trauma and aggression and that these global network features complicate the manifestation of aggression. These small-world properties reflect the overall efficiency and integration of the brain network, with their disruption potentially altering global functional connectivity.^22,41^ These masking effects presented by the small-world properties suggest that the global network organization may act as a broader modulatory factor, indirectly influencing aggression by altering how different brain regions communicate and integrate information. While global network topology may not directly cause aggression, it appears to influence it through its impact on the connectivity between regions involved in emotional regulation and cognitive control.

## Limitations

Several limitations of this study warrant consideration. First, we did not directly assess participants’ IQ or years of education; to minimize these differences, all adolescents were recruited from the same school grades, but future work should include standardized measures of cognitive ability and educational attainment. Second, although the CTQ-SF distinguishes five trauma subtypes (emotional abuse, physical abuse, sexual abuse, emotional neglect, physical neglect), our sample size did not allow us to examine how specific subtypes uniquely relate to aggressive behaviour; larger cohorts are needed to parse these effects. Finally, the cross-sectional design precludes causal inference—while we observed that childhood maltreatment is associated with reactive aggression and altered network topology, longitudinal studies are required to determine the directionality of these relationships.

## Conclusion

In conclusion, this study demonstrates that some important brain functional network topological biomarkers, including small-world properties and the nodal efficiency of the left SPG, play key mediating roles in the relationship between childhood abuse and RA in IGD adolescents. These findings highlight the complex neurobiological mechanisms underlying aggression, where both local brain dysfunctions and global network topological disruptions contribute to aggressive behaviour. These findings offer valuable guidance for future intervention strategies since improving the integration and efficiency of brain functional networks, particularly in regions involving cognitive control and emotional regulation, may help reduce aggression in youths with IGD and childhood maltreatment.

## Supplementary Material

fcaf407_Supplementary_Data

## Data Availability

De-identified raw data underlying this study are available from the corresponding author upon reasonable request, subject to approval by the local ethics committee and compliance with applicable regulations. A minimal MATLAB snippet used for FDR correction in the correlation analyses is provided in the Supplementary materials. All other analyses were conducted using off-the-shelf software: DPABI v6.0 (http://rfmri.org/dpabi) for preprocessing, GRETNA (http://www.nitrc.org/projects/gretna/) for graph-theoretical analyses, and the PROCESS macro for SPSS v23 (https://www.processmacro.org/download.html) for mediation analysis.

## References

[1] First MB . Diagnostic and statistical manual of mental disorders, 5th edition, and clinical utility. J Nerv Ment Dis. 2013;201(9):727–729. 23995026 10.1097/NMD.0b013e3182a2168a

[2] Evren C, Evren B, Dalbudak E, Topcu M, Kutlu N. Relationships of internet addiction and internet gaming disorder symptom severities with probable attention deficit/hyperactivity disorder, aggression and negative affect among university students. Atten Defic Hyperact Disord. 2019;11(4):413–421. 31062235 10.1007/s12402-019-00305-8

[3] Yu H, Cho J. Prevalence of internet gaming disorder among Korean adolescents and associations with non-psychotic psychological symptoms, and physical aggression. Am J Health Behav. 2016;40(6):705–716. 27779939 10.5993/AJHB.40.6.3

[4] Chen SJ, Hong HW, Zhou YH, Huang XY, Gao XM. Neurobiological correlates of reactive aggression in young adults with internet gaming disorder. Brain Res Bull. 2025:220:111133. 39571624 10.1016/j.brainresbull.2024.111133

[5] Deng X, Hu YB, Liu CY, et al Psychological distress and aggression among adolescents with internet gaming disorder symptoms. Psychiatry Res. 2024:331:115624. 38039647 10.1016/j.psychres.2023.115624

[6] Zhang CY, Zhang Q, Wang SS, Xu W. Childhood trauma and aggression among Chinese college students: The mediation of self-compassion and moderation of left-behind experience. Psychol Trauma. 2023;15:S73–S81. 36913294 10.1037/tra0001452

[7] Zaorska J, Kopera M, Trucco EM, Suszek H, Kobylinski P, Jakubczyk A. Childhood trauma, Emotion regulation, and pain in individuals with alcohol use disorder. Front Psychiatry. 2020;11:554150. 10.3389/fpsyt.2020.554150 PMC7661433 33192667

[8] Liu Q, Ouyang LJ, Fan LJ, et al Association between childhood trauma and internet gaming disorder: A moderated mediation analysis with depression as a mediator and psychological resilience as a moderator. BMC Psychiatry. 2024;24(1):412. 38834952 10.1186/s12888-024-05863-4 PMC11151498

[9] Delhove M, Greitemeyer T. Can violent video game-related aggression spread to others? Effects on retaliatory and displaced aggression. Int Rev Soc Psychol. 2019;32(1):14.

[10] Wang LL, Yin Y, Feng W, et al Childhood trauma and cognitive deficits in patients with schizophrenia: Mediation by orbitofrontal cortex H-shaped sulci volume. J Psychiatry Neurosci. 2022;47(3):E209–E217. 35654451 10.1503/jpn.210178 PMC9177195

[11] Liu GC, Yen JY, Chen CY, et al Brain activation for response inhibition under gaming cue distraction in internet gaming disorder. Kaohsiung J Med Sci. 2014;30(1):43–51. 24388058 10.1016/j.kjms.2013.08.005 PMC11916293

[12] Zhang JL, Chen SY, Jiang Q, et al Disturbed craving regulation to gaming cues in internet gaming disorder: Implications for uncontrolled gaming behaviors. J Psychiatr Res. 2021;140:250–259. 34119910 10.1016/j.jpsychires.2021.05.051

[13] Dauvermann MR, Mothersill D, Rokita KI, et al Changes in default-mode network associated with childhood trauma in schizophrenia. Schizophr Bull. 2021;47(5):1482–1494. 33823040 10.1093/schbul/sbab025 PMC8379545

[14] Paz-Alonso PM, Navalpotro-Gomez I, Boddy P, et al Functional inhibitory control dynamics in impulse control disorders in Parkinson's disease. Mov Disord. 2020;35(2):316–325. 31710401 10.1002/mds.27885

[15] Wang ZL, Song KR, Zhou N, Potenza MN, Zhang JT, Dong GH. Gender-related differences in involvement of addiction brain networks in internet gaming disorder: Relationships with craving and emotional regulation. Prog Neuropsychopharmacol Biol Psychiatry. 2022;118:118110574. 10.1016/j.pnpbp.2022.110574 35569619

[16] Chun JW, Park CH, Kim JY, et al Altered core networks of brain connectivity and personality traits in internet gaming disorder. J Behav Addict. 2020;9(2):298–311. 32592635 10.1556/2006.2020.00014 PMC8939405

[17] Wang ZL, Potenza MN, Song KR, Dong GH, Fang XY, Zhang JT. Subgroups of internet gaming disorder based on addiction-related resting-state functional connectivity. Addiction. 2023;118(2):327–339. 36089824 10.1111/add.16047

[18] Kjærstad HL, Macoveanu J, Knudsen GM, et al Neural responses during down-regulation of negative emotion in patients with recently diagnosed bipolar disorder and their unaffected relatives. Psychol Med. 2023;53(4):1254–1265. 37010225 10.1017/S0033291721002737

[19] Dong G, Lin X, Zhou H, Lu Q. Cognitive flexibility in internet addicts: FMRI evidence from difficult-to-easy and easy-to-difficult switching situations. Addict Behav. 2014;39(3):677–683. 24368005 10.1016/j.addbeh.2013.11.028

[20] Hong SB, Harrison BJ, Dandash O, et al A selective involvement of putamen functional connectivity in youth with internet gaming disorder. Brain Res. 2015;1602:85–95. 25553620 10.1016/j.brainres.2014.12.042

[21] Park CH, Chun JW, Cho H, Jung YC, Choi J, Kim DJ. Is the internet gaming-addicted brain close to be in a pathological state? Addict Biol. 2017;22(1):196–205. 26135331 10.1111/adb.12282

[22] Wang ZL, Liu XY, Hu YB, Zheng H, Du XX, Dong GH. Altered brain functional networks in internet gaming disorder: Independent component and graph theoretical analysis under a probability discounting task. CNS Spectr. 2019;24(5):544–556. 30968814 10.1017/S1092852918001505

[23] Zhang ZJ, Wang SZ, Du XX, Qi YY, Wang LX, Dong GH. Brain responses to positive and negative events in individuals with internet gaming disorder during real gaming. J Behav Addict. 2023;12(3):758–774. 37651282 10.1556/2006.2023.00039 PMC10562809

[24] Çakar S, Eren G. Internet addiction in constipated adolescents. J Gastroenterol. 2023;34(3):287–292. 10.5152/tjg.2023.22190 PMC10152157 36919833

[25] Wang X, Ding FJ, Cheng C, He JY, Wang X, Yao SQ. Psychometric properties and measurement invariance of the childhood trauma questionnaire (short form) across genders, time points and presence of Major depressive disorder among Chinese adolescents. Front Psychol. 2022;13:816051. 35478747 10.3389/fpsyg.2022.816051 PMC9036057

[26] Raine A, Dodge K, Loeber R, et al The reactive-proactive aggression questionnaire: Differential correlates of reactive and proactive aggression in adolescent boys. Aggress Behav. 2006;32(2):159–171. 20798781 10.1002/ab.20115 PMC2927832

[27] Jenkinson M, Bannister P, Brady M, Smith S. Improved optimization for the robust and accurate linear registration and motion correction of brain images. Neuroimage. 2002;17(2):825–841. 12377157 10.1016/s1053-8119(02)91132-8

[28] Ashburner J . A fast diffeomorphic image registration algorithm. Neuroimage. 2007;38(1):95–113. 17761438 10.1016/j.neuroimage.2007.07.007

[29] Friston KJ, Williams S, Howard R, Frackowiak RSJ, Turner R. Movement-related effects in fMRI time-series. Magn Reson Med. 1996;35(3):346–355. 8699946 10.1002/mrm.1910350312

[30] He Y, Wang J, Wang L, et al Uncovering intrinsic modular organization of spontaneous brain activity in humans. PLoS One. 2009;4(4):e5226. 19381298 10.1371/journal.pone.0005226 PMC2668183

[31] Wang J, Wang X, Xia M, Liao X, Evans A, He Y. GRETNA: A graph theoretical network analysis toolbox for imaging connectomics. Front Hum Neurosci. 2015;9:386. 26175682 10.3389/fnhum.2015.00386 PMC4485071

[32] Wen H, Liu Y, Rekik I, et al Combining disrupted and discriminative topological properties of functional connectivity networks as neuroimaging biomarkers for accurate diagnosis of early Tourette syndrome children. Mol Neurobiol. 2018;55(4):3251–3269. 28478510 10.1007/s12035-017-0519-1

[33] Xin H, Liang C, Fu Y, et al Disrupted brain structural networks associated with depression and cognitive dysfunction in cerebral small vessel disease with microbleeds. Prog Neuropsychopharmacol Biol Psychiatry. 2024;131:110944. 38246218 10.1016/j.pnpbp.2024.110944

[34] Gao Y, Wang S, Xin H, et al Disrupted gray matter networks associated with cognitive dysfunction in cerebral small vessel disease. Brain Sci. 2023;13(10):1359. 37891728 10.3390/brainsci13101359 PMC10605932

[35] Benjamini Y, Hochberg Y. Controlling the false discovery rate: A practical and powerful approach to multiple testing. J R Stat Soc Ser B: Methodol. 1995;57(1):289–300.

[36] Hayes AF. PROCESS: A versatile computational tool for observed variable mediation, moderation, and conditional process modeling. University of Kansas, KS. 2008. http://www.afhayes.com/public/process2012.pdf

[37] Shrout PE, Bolger N. Mediation in experimental and nonexperimental studies: New procedures and recommendations. Psychol Methods. 2002;7(4):422–445. 12530702

[38] Preacher KJ, Hayes AF. Asymptotic and resampling strategies for assessing and comparing indirect effects in multiple mediator models. Behav Res Methods. 2008;40(3):879–891. 18697684 10.3758/brm.40.3.879

[39] Mackinnon DP . Introduction to statistical mediation analysis. Lawrence Erlbaum Associates; 2008.

[40] Wen ZL, Ye BJ. Analyses of mediating effects: The development of methods and models. Adv Psychol Ence. 2014;22(5):731–745.

[41] Zhai JQ, Luo L, Qiu LJ, et al The topological organization of white matter network in internet gaming disorder individuals. Brain Imaging Behav. 2017;11(6):1769–1778. 27815774 10.1007/s11682-016-9652-0

[42] Wang LX, Wu LD, Lin X, et al Altered brain functional networks in people with internet gaming disorder: Evidence from resting-state fMRI. Psychiatry Res Neuroimaging. 2016;254:156–163. 27447451 10.1016/j.pscychresns.2016.07.001

[43] Yin FRZ . The physical, psychological, and social consequences of adolescents with internet addiction. Dissertation/thesis. 2009.

[44] Botchway E, Kooper CC, Pouwels PJW, et al Resting-state network organisation in children with traumatic brain injury. Cortex. 2022;154:89–104. 35763900 10.1016/j.cortex.2022.05.014

[45] Xu J, Chen FQ, Lei D, et al Disrupted functional network topology in children and adolescents with post-traumatic stress disorder. Front Neurosci. 2018;12:709. 30356635 10.3389/fnins.2018.00709 PMC6189287

[46] Wolbers T, Weiller C, Büchel C. Contralateral coding of imagined body parts in the superior parietal lobe. Cerebral Cortex. 2003;13(4):392–399. 12631568 10.1093/cercor/13.4.392

[47] Corbetta M, Shulman G. Control of goal-directed and stimulus-driven attention in the brain. Nat Rev Neurosci. 2002;3:201–215. 11994752 10.1038/nrn755

[48] Coccaro EF, Sripada CS, Yanowitch RN, Phan KL. Corticolimbic function in impulsive aggressive behavior. Biol Psychiatry. 2011;69(12):1153–1159. 21531387 10.1016/j.biopsych.2011.02.032

[49] Won GH, Bae S, Kim HK, Choi TY. Subcortical volume analysis in non-suicidal self-injury adolescents: A pilot study. Psychiatry Res Neuroimaging. 2023;331:111617. 36907098 10.1016/j.pscychresns.2023.111617

[50] Janiri D, Sani G, De Rossi P, et al Hippocampal subfield volumes and childhood trauma in bipolar disorders. J Affect Disord. 2019;253:35–43. 31022627 10.1016/j.jad.2019.04.071

[51] Dodge KA, Coie JD. Social-information-processing factors in reactive and proactive aggression in childrens peer groups. J Pers Soc Psychol. 1987;53(6):1146–1158. 3694454 10.1037//0022-3514.53.6.1146

[52] Prossin AR, Love TM, Koeppe RA, Zubieta JK, Silk KR. Dysregulation of regional endogenous opioid function in borderline personality disorder. Am J Psychiatry. 2010;167(8):925–933. 20439388 10.1176/appi.ajp.2010.09091348 PMC6863154

[53] Chen JR, Luo QY, Li YH, et al Intrinsic brain abnormalities in female major depressive disorder patients with childhood trauma: A resting-state functional magnetic resonance imaging study. Front Neurosci. 2022;16:930997. 36017185 10.3389/fnins.2022.930997 PMC9395929

