# Supplementary material for: Disrupted functional network topology mediates the correlation between childhood trauma and aggression in youths with internet gaming disorder
Source: Brain Commun. 2025 Oct 16;7(6):fcaf407. doi: 10.1093/braincomms/fcaf407 (PMC12596128; doi:10.1093/braincomms/fcaf407)
Supplement: fcaf407_Supplementary_Data [file fcaf407_supplementary_data.docx]

**Supplementary Materials**

Supplementary Table 1. **Regions of interests (ROIs) from automated anatomical labeling (AAL) atlas and the corresponding functional modules.**

| Abbr. | Regions | Functional module |
| --- | --- | --- |
| PreCG.L | Precentral gyrus (Left) | auditory/motor |
| PreCG.R | Precentral gyrus (Right) | auditory/motor |
| SFGdor.L | Superior frontal gyrus, dorsolateral (Left) | DMN |
| SFGdor.R | Superior frontal gyrus, dorsolateral (Right) | DMN |
| ORBsup.L | Superior frontal gyrus, orbital part (Left) | attention |
| ORBsup.R | Superior frontal gyrus, orbital part (Right) | DMN |
| MFG.L | Middle frontal gyrus (Left) | attention |
| MFG.R | Middle frontal gyrus (Right) | attention |
| ORBmid.L | Middle frontal gyrus orbital part (Left) | attention |
| ORBmid.R | Middle frontal gyrus orbital part (Right) | attention |
| IFGoperc.L | Inferior frontal gyrus, opercular part (Left) | attention |
| IFGoperc.R | Inferior frontal gyrus, opercular part (Right) | attention |
| IFGtriang.L | Inferior frontal gyrus, triangular part (Left) | attention |
| IFGtriang.R | Inferior frontal gyrus, triangular part (Right) | attention |
| ORBinf.L | Inferior frontal gyrus, orbital part (Left) | attention |
| ORBinf.R | Inferior frontal gyrus, orbital part (Right) | attention |
| ROL.L | Rolandic operculum (Left) | auditory/motor |
| ROL.R | Rolandic operculum (Right) | auditory/motor |
| SMA.L | Supplementary motor area (Left) | attention |
| SMA.R | Supplementary motor area (Right) | auditory/motor |
| OLF.L | Olfactory cortex (Left) | Subcortical |
| OLF.R | Olfactory cortex (Right) | Subcortical |
| SFGmed.L | Superior frontal gyrus, medial (Left) | DMN |
| SFGmed.R | Superior frontal gyrus, medial (Right) | DMN |
| ORBsupmed.L | Superior frontal gyrus, medial orbital (Left) | DMN |
| ORBsupmed.R | Superior frontal gyrus, medial orbital (Right) | DMN |
| REC.L | Gyrus rectus (Left) | DMN |
| REC.R | Gyrus rectus (Right) | DMN |
| INS.L | Insula (Left) | auditory/motor |
| INS.R | Insula (Right) | auditory/motor |
| ACG.L | Anterior cingulate and paracingulate gyri (Left) | DMN |
| ACG.R | Anterior cingulate and paracingulate gyri (Right) | DMN |
| DCG.L | Median cingulate and paracingulate gyri (Left) | Subcortical |
| DCG.R | Median cingulate and paracingulate gyri (Right) | Subcortical |
| PCG.L | Posterior cingulate gyrus (Left) | DMN |
| PCG.R | Posterior cingulate gyrus (Right) | DMN |
| HIP.L | Hippocampus (Left) | Subcortical |
| HIP.R | Hippocampus (Right) | Subcortical |
| PHG.L | Parahippocampal gyrus (Left) | Subcortical |
| PHG.R | Parahippocampal gyrus (Right) | Subcortical |
| AMYG.L | Amygdala (Left) | Subcortical |
| AMYG.R | Amygdala (Right) | Subcortical |
| CAL.L | Calcarine fissure and surrounding cortex (Left) | vision |
| CAL.R | Calcarine fissure and surrounding cortex (Right) | vision |
| CUN.L | Cuneus (Left) | vision |
| CUN.R | Cuneus (Right) | vision |
| LING.L | Lingual gyrus (Left) | vision |
| LING.R | Lingual gyrus (Right) | vision |
| SOG.L | Superior occipital gyrus (Left) | vision |
| SOG.R | Superior occipital gyrus (Right) | vision |
| MOG.L | Middle occipital gyrus (Left) | vision |
| MOG.R | Middle occipital gyrus (Right) | vision |
| IOG.L | Inferior occipital gyrus (Left) | vision |
| IOG.R | Inferior occipital gyrus (Right) | vision |
| FFG.L | Fusiform gyrus (Left) | vision |
| FFG.R | Fusiform gyrus (Right) | vision |
| PoCG.L | Postcentral gyrus (Left) | auditory/motor |
| PoCG.R | Postcentral gyrus (Right) | auditory/motor |
| SPG.L | Superior parietal gyrus (Left) | auditory/motor |
| SPG.R | Superior parietal gyrus (Right) | auditory/motor |
| IPL.L | Inferior parietal, but supramarginal and angular gyri (Left) | attention |
| IPL.R | Inferior parietal, but supramarginal and angular gyri (Right) | attention |
| SMG.L | Supramarginal gyrus (Left) | auditory/motor |
| SMG.R | Supramarginal gyrus (Right) | auditory/motor |
| ANG.L | Angular gyrus (Left) | attention |
| ANG.R | Angular gyrus (Right) | attention |
| PCUN.L | Precuneus (Left) | DMN |
| PCUN.R | Precuneus (Right) | DMN |
| PCL.L | Paracentral lobule (Left) | auditory/motor |
| PCL.R | Paracentral lobule (Right) | auditory/motor |
| CAU.L | Caudate nucleus (Left) | Subcortical |
| CAU.R | Caudate nucleus (Right) | Subcortical |
| PUT.L | Lenticular nucleus, putamen (Left) | Subcortical |
| PUT.R | Lenticular nucleus, putamen (Right) | Subcortical |
| PAL.L | Lenticular nucleus, pallidum (Left) | Subcortical |
| PAL.R | Lenticular nucleus, pallidum (Right) | Subcortical |
| THA.L | Thalamus (Left) | Subcortical |
| THA.R | Thalamus (Right) | Subcortical |
| HES.L | Heschl gyrus (Left) | auditory/motor |
| HES.R | Heschl gyrus (Right) | auditory/motor |
| STG.L | Superior temporal gyrus (Left) | auditory/motor |
| STG.R | Superior temporal gyrus (Right) | auditory/motor |
| TPOsup.L | Temporal pole: superior temporal gyrus (Left) | attention |
| TPOsup.R | Temporal pole: superior temporal gyrus (Right) | auditory/motor |
| MTG.L | Middle temporal gyrus (Left) | DMN |
| MTG.R | Middle temporal gyrus (Right) | DMN |
| TPOmid.L | Temporal pole: middle temporal gyrus (Left) | Subcortical |
| TPOmid.R | Temporal pole: middle temporal gyrus (Right) | Subcortical |
| ITG.L | Inferior temporal gyrus (Left) | attention |
| ITG.R | Inferior temporal gyrus (Right) | DMN |

Note: The functional modular division of brain regions was based on a previous study ([He](#_ENREF_1) *[et al.](#_ENREF_1)*[, 2009](#_ENREF_1)).

Supplementary Table 2. **Global and nodal topological properties used in the study.**

| **Global properties** | **General descriptions** |
| --- | --- |
| Shortest path length (L_p_) | L_p_ is defined as the average length of the shortest path between every two nodes in network G, which quantifies the ability for information to be propagated in parallel, which is computed as follows:  $L_{P}\left( G \right)=\frac{1}{N(N-1)}\sum_{i\neq j\in G} L_{\mathrm{ij}}$ |
| Global Efficiency (E_glob_) | E_glob_ is defined as the mean value of all regions’ global efficiency, which is computed as follows:  $E_{\mathrm{glob}}\left( G \right)=\frac{1}{N(N-1)}\sum_{i\neq j\in G} \frac{1}{L_{\mathrm{ij}}}$ |
| Local Efficiency (E_loc_) | E_loc_ is defined as the mean value of all regions’ local efficiency, is computed as follows:  $E_{\mathrm{loc}}\left( G \right)=\frac{1}{N}\sum_{i\in G} E_{\mathrm{glob}}(G_{i})$  where G_i_ denotes the subgraph composed of the nearest neighbors of node i. |
| Clustering coefficient (C_p_) | C_p_ is the average clustering coefficient over all nodes, which indicates the extent of local interconnectivity or cliquishness in a network, which is computed as follows:  $\text{C}_{\text{i}}\text{=}\frac{\text{2}}{\text{k}_{\text{i}}\text{(k}_{\text{i}}\text{-1)}}\sum_{j,k} {(\bar{w_{\text{ij}}} \bar{w_{\text{jk}}} \bar{w_{\text{ki}}})}^{1/3}$  where k_i_ is the degree of node i, and $\bar{w}$ is the weight, which is scaled by the mean of all weights to control each subject’s cost at the same level. |
| Normalized L_p_ (λ) | λ=L_p_^real^/ L_p_^rand^, L_p_^rand^ is the mean shortest path length of 100 matched random networks. |
| Normalized C_p_ (γ) | γ= C_p_^real^/ C_p_^rand^, C_p_^rand^ is the mean clustering coefficient of 100 matched random networks. |
| Small-worldness σ | σ=γ/λ, A real network would be considered small world if γ>1 and λ≈1. |
| **Nodal properties** | **General descriptions** |
| Nodal Efficiency E_nodal_(i) | E_nodal_(i) measure the average shortest path length between this node and all the other nodes in the network, which is computed as follows:  $E_{\mathrm{nodal}}\left( i \right)=\frac{1}{N-1}\sum_{i\neq j\in G} \frac{1}{L_{\mathrm{ij}}}$ |

Code used in the manuscript:

% False discovery rate correction was applied to the p-values from the correlation analysis.

FDR=mafdr(p,‘BHFDR’,true);

**Supplementary references**

**He, Y., Wang, J., Wang, L., Chen, Z. J., Yan, C., Yang, H., Tang, H., Zhu, C., Gong, Q. & Zang, Y.** (2009). Uncovering intrinsic modular organization of spontaneous brain activity in humans. *PloS one* **4**, e5226.
